# Supplementary material for: Whole genome sequence analysis reveals the broad distribution of the RtxA type 1 secretion system and four novel putative type 1 secretion systems throughout the Legionella genus
Source: PLoS One. 2020 Jan 14;15(1):e0223033. doi: 10.1371/journal.pone.0223033 (PMC6959600; doi:10.1371/journal.pone.0223033)
Supplement: S1 File — Supplementary figures and tables. PONE-D-19-25375R1_Supplementary_Information. (DOCX) [file pone.0223033.s001.docx]

Full title: Whole genome sequence analysis reveals broad distribution of the RtxA type 1 secretion system and four novel type 1 secretion systems throughout the *Legionella* genus

Short title: Type 1 secretion systems among *Legionella* species

Authors: Connor L. Brown^1,2^, Emily Garner^1,3^, Guillaume Jospin^4^, David A. Coil^4^, David O. Schwake^5^, Jonathan A. Eisen^4,6^, Biswarup Mukhopadhyay^2^ & Amy J. Pruden^1^*

^1^Via Department of Civil and Environmental Engineering, Virginia Tech, Blacksburg, VA 24061, USA

^2^Department of Biochemistry, Virginia Tech, Blacksburg, VA 24061, USA

^3^Department of Civil and Environmental Engineering, West Virginia University, Morgantown, WV 26506, USA

^4^Genome Center, University of California, Davis, CA 95617, USA

^5^Department of Natural Sciences, Middle Georgia State University, Macon, GA 31204, USA

^6^Evolution and Ecology, Medical Microbiology and Immunology, University of California, Davis, CA 95167, USA

Corresponding Author (e-mail: apruden@vt.edu)

| Query Species | Gene name | Score | % Identity | Subject Length | Query Length | Strand | Expect | Gaps | GenBank Query Info |
| --- | --- | --- | --- | --- | --- | --- | --- | --- | --- |
| L. erythra | RecA | 542 | 403/458 (88%) | 1041 | 463 | Plus/Plus | 1.00E-149 | 0 | >Legionella erythra strain MDC1578 recombinase A protein (recA) gene, partial cds |
| L. erythra | mip | 941 | 655/728 (90%) | 741 | 728 | Plus/Plus | 0 | 0 | >Legionella erythra macrophage infectivity potentiator (mip) gene, complete cds |
| L. erythra | 16S | 2599 | 1441/1457 (99%) | 1513 | 1457 | Plus/Plus | 0 | 0 | >Legionella erythra strain SE-32A-C8 16S ribosomal RNA gene, partial sequence |
| L. erythra | proA | 656 | 464/518 (90%) | 517 | 518 | Plus/Plus | 0 | 0 | >Legionella erythra strain ATCC 35303 ProA (proA) gene, partial cds |
| L. rubrilucens | 16S | 2617 | 1446/1568 (99%) | 1513 | 1468 | Plus/Plus | 0.00E+00 | 0 | >ENA\|X73398\|X73398.1 L.rubrilucens (ATCC 35304) gene for 16S rRNA |
| L. rubrilucens | 16S | 2638 | 1450/1460 (99%) | 1513 | 1460 | Plus/Plus | 0 | 4/1460 | >ENA\|Z32643\|Z32643.1 L.rubrilucens (WA-270A-C2) gene for 16S ribosomal RNA |
| L. rubrilucens | gyrB | 1219 | 784/846 (93%) | 2418 | 846 | Plus/Plus | 0 | 0 | >ENA\|Z24716\|Z24716.1 L.rubrilucens 23S and 5S rRNA genes, partial CDS&apos;s. |
| L. rubrilucens | mip | 1208 | 712/714 (96%) | 741 | 747 | Plus/Plus | 0 | 0 | >lcl\|U92218.1_cds_AAC45704.1_1 [gene=mip] [protein=macrophage infectivity potentiator] [protein_id=AAC45704.1] [location=69..815] [gbkey=CDS] |
| L. rubrilucens | proA | 833 | 495/517 (96%) | 1642 | 517 | Plus/Plus | 0 | 0 | >ENA\|JN086211\|JN086211.1 Legionella rubrilucens strain ATCC 35304 ProA (proA) gene, partial cds |
| L. rubrilucens | recA | 750 | 444/463 (96%) | 1041 | 463 | Plus/Plus | 0 | 0 | >ENA\|JF720513\|JF720513.1 Legionella rubrilucens strain MDC1576 recombinase A protein (recA) gene, partial cds |
| L. rubrilucens | rpoB | 571 | 359/384 (93%) | 4023 | 384 | Plus/Plus | 1.00E-166 | 0 | >ENA\|JF720620\|JF720620.1 Legionella rubrilucens strain MDC1576 RNA polymerase beta subunit (rpoB) gene, partial cds. |
| L. taurensis | 16S | 2748 | 1490/1491 (99%) | 1513 | 1491 | Plus/Plus | 0.00E+00 | 0 | >ENA\|DQ667196\|DQ667196.1 Legionella taurinensis strain ATCC 700508 16S ribosomal RNA gene, complete sequence. |
| L. taurensis | gyrB | 750 | 407/408 (99%) | 2418 | 408 | Plus/Plus | 0 |  | >Legionella taurinensis strain MDC1603 DNA gyrase subunit B (gyrB) gene, partial cds |
| L. taurensis | mip | 1192 | 645/645 (100%) | 741 | 645 | Plus/Plus | 0 | 0 | >ENA\|AF022342\|AF022342.2 Legionella taurinensis macrophage infectivity potentiator protein (mip) gene, partial cds. |
| L. taurensis | proA | 955 | 517/517 (100%) | 1642 | 517 | Plus/Plus | 0 | 0 | >ENA\|JN086210\|JN086210.1 Legionella taurinensis strain ATCC 700508 ProA (proA) gene, partial cds. |
| L. taurensis | recA | 836 | 463/463 (100%) | 1041 | 463 | Plus/Plus | 0 | 0 | >Legionella taurinensis strain MDC1603 recombinase A protein (recA) gene, partial cds |
| L. taurensis | rpoB | 532 | 296/300 (99%) | 4023 | 300 | Plus/Plus | 4.00E-155 | 0 | >ENA\|AY883051\|AY883051.1 Legionella taurinensis RNA polymerase beta (rpoB) gene, partial cds. |

**Table A.** Blastn sequence analysis of conserved loci from the novel *Legionella taurinensis* strain reveal similarity between the novel strains and previously sequenced loci in *L. taurinensis.*

^
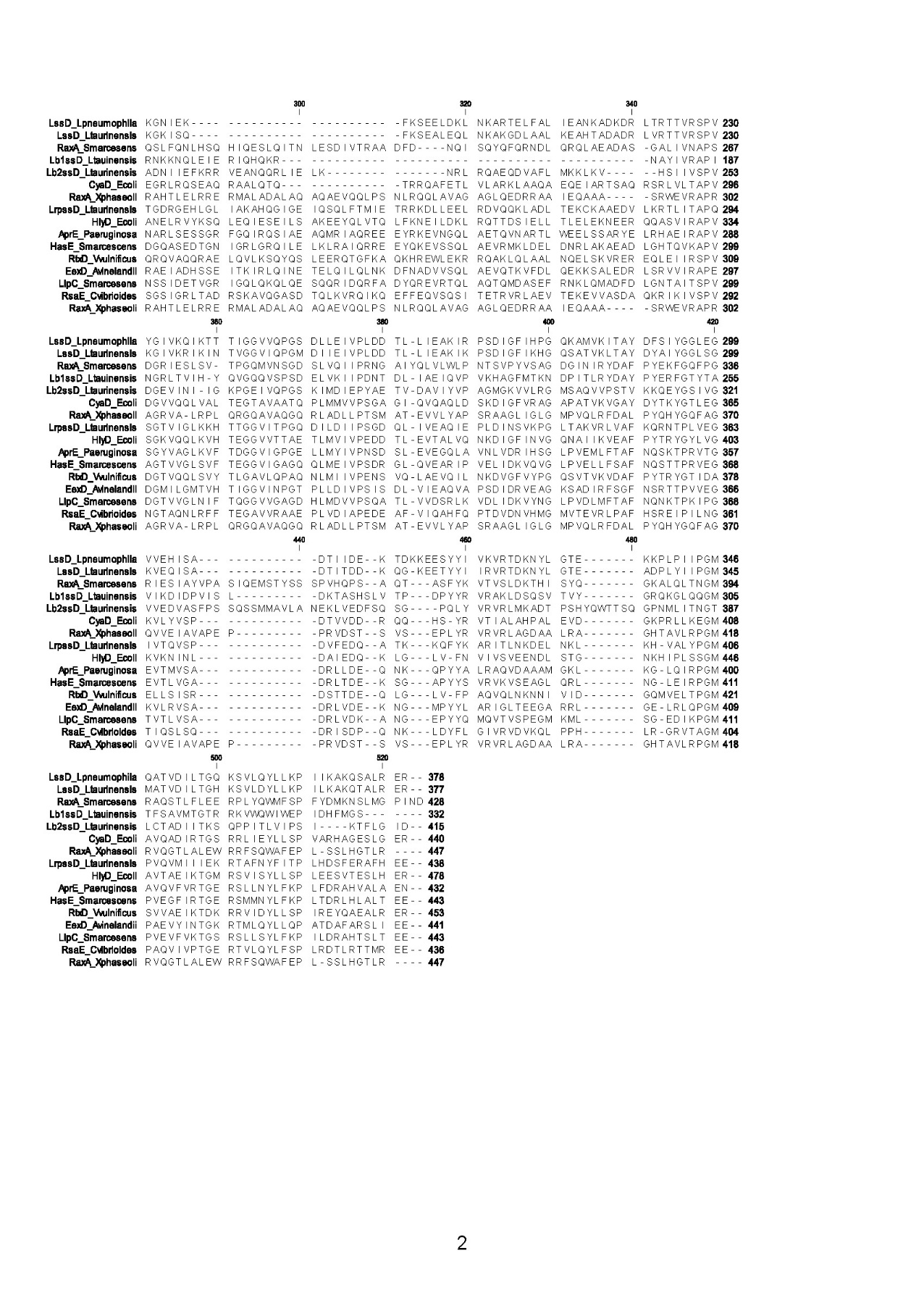
^*
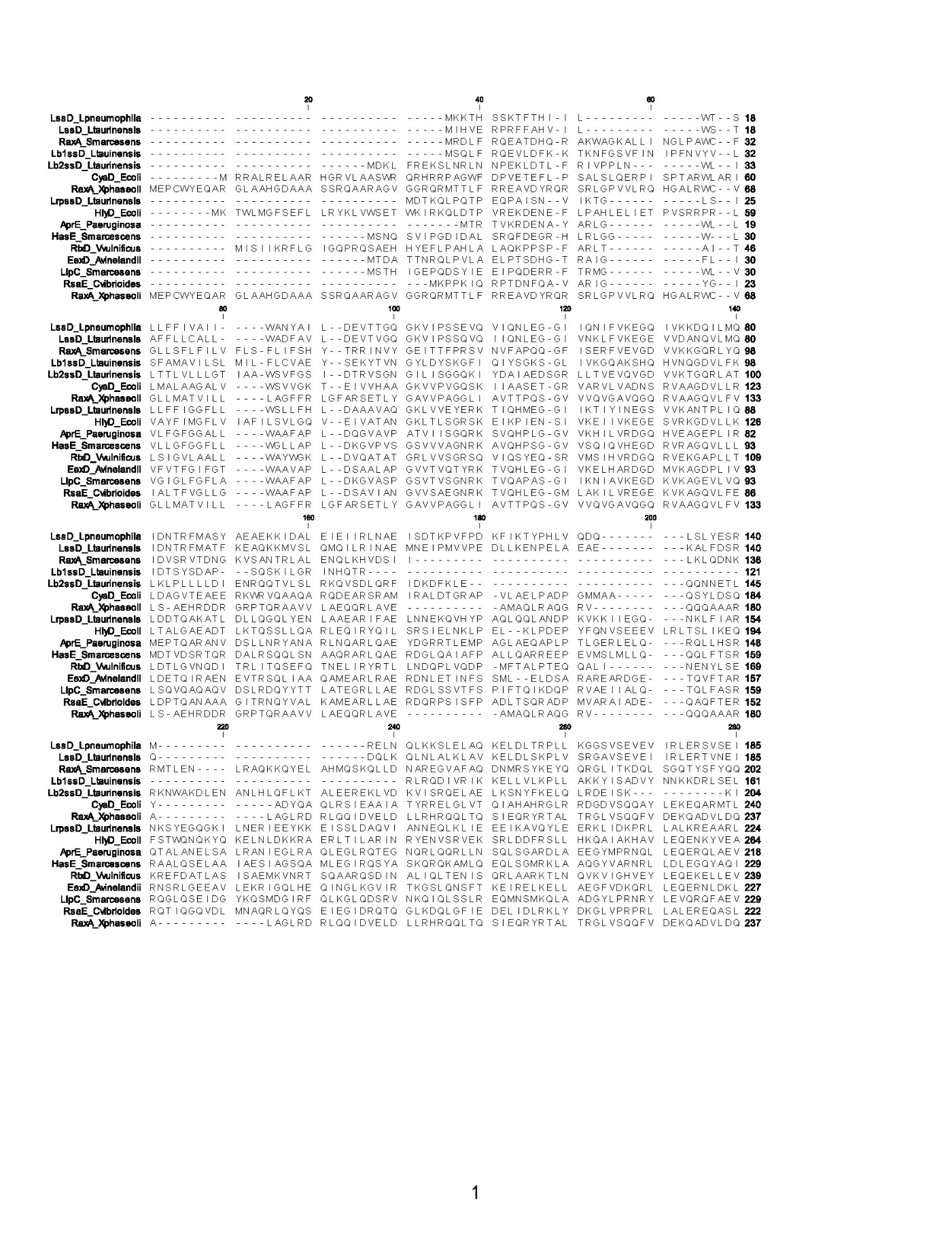
*

**Figure A.** Alignment of membrane fusion proteins from type 1 secretion system (T1SSs) of diverse functions. *L. pneumophila* LssD is 60% identical to *L. taurinensis* LssD.


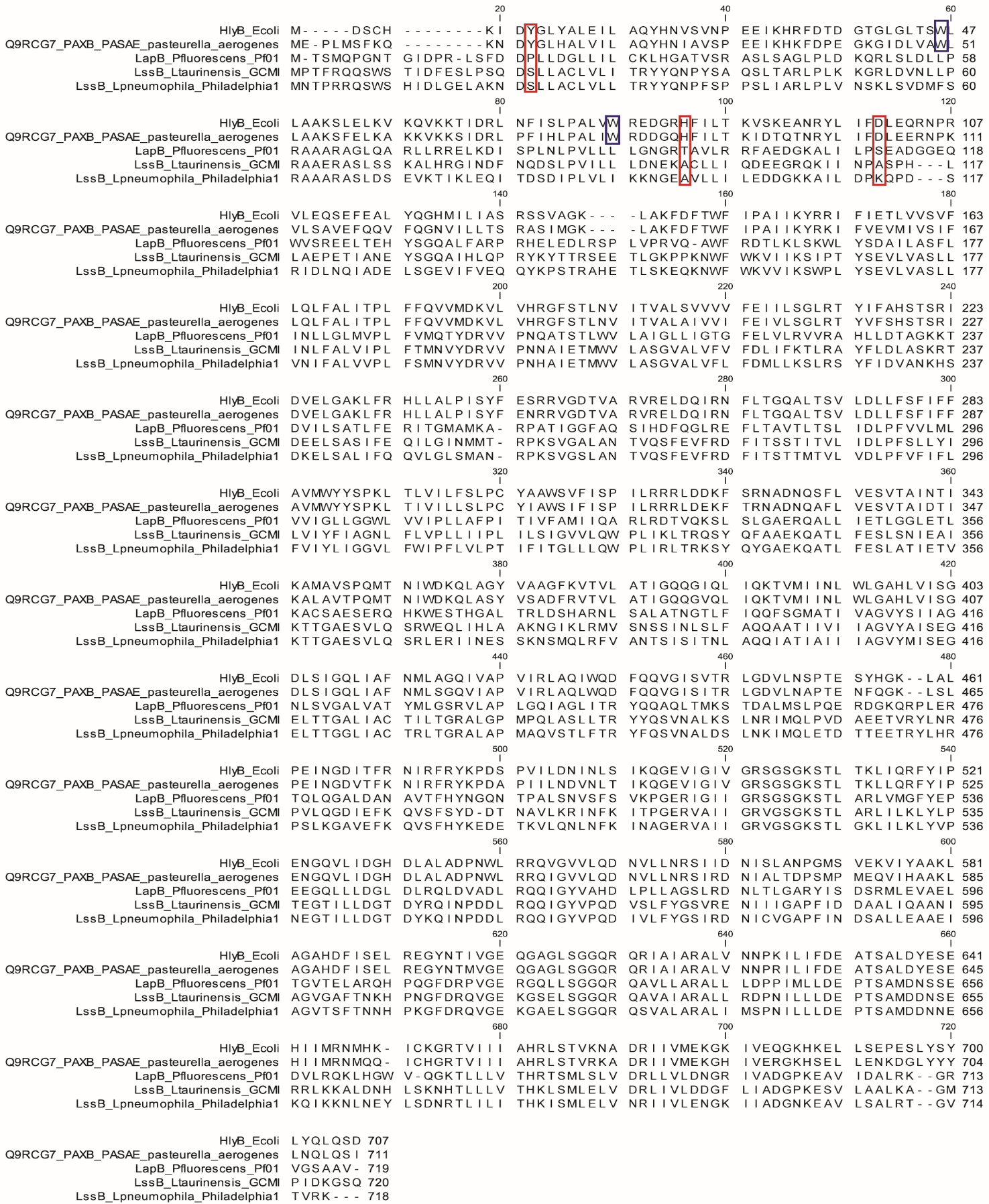


**Figure B.** Alignment of CLD-type type 1 secretion system (T1SS) ABC transporters, including *Legionella pneumophila* LssB and the identified LssB homolog in *L. taurinensis. L. pneumophila* LssB is 60% identical to *L. taurinensis* LssB. *L. taurinensis* and *L. pneumophila* LssB possess the LapB-type CLD, while HlyB from *E. coli* and *P. aerogenes* possess the HlyB-type CLD, consistent with previous observations [1, 2].





**Figure C.** Unmodified RAxML tree (Figure 3 of the main text).

**
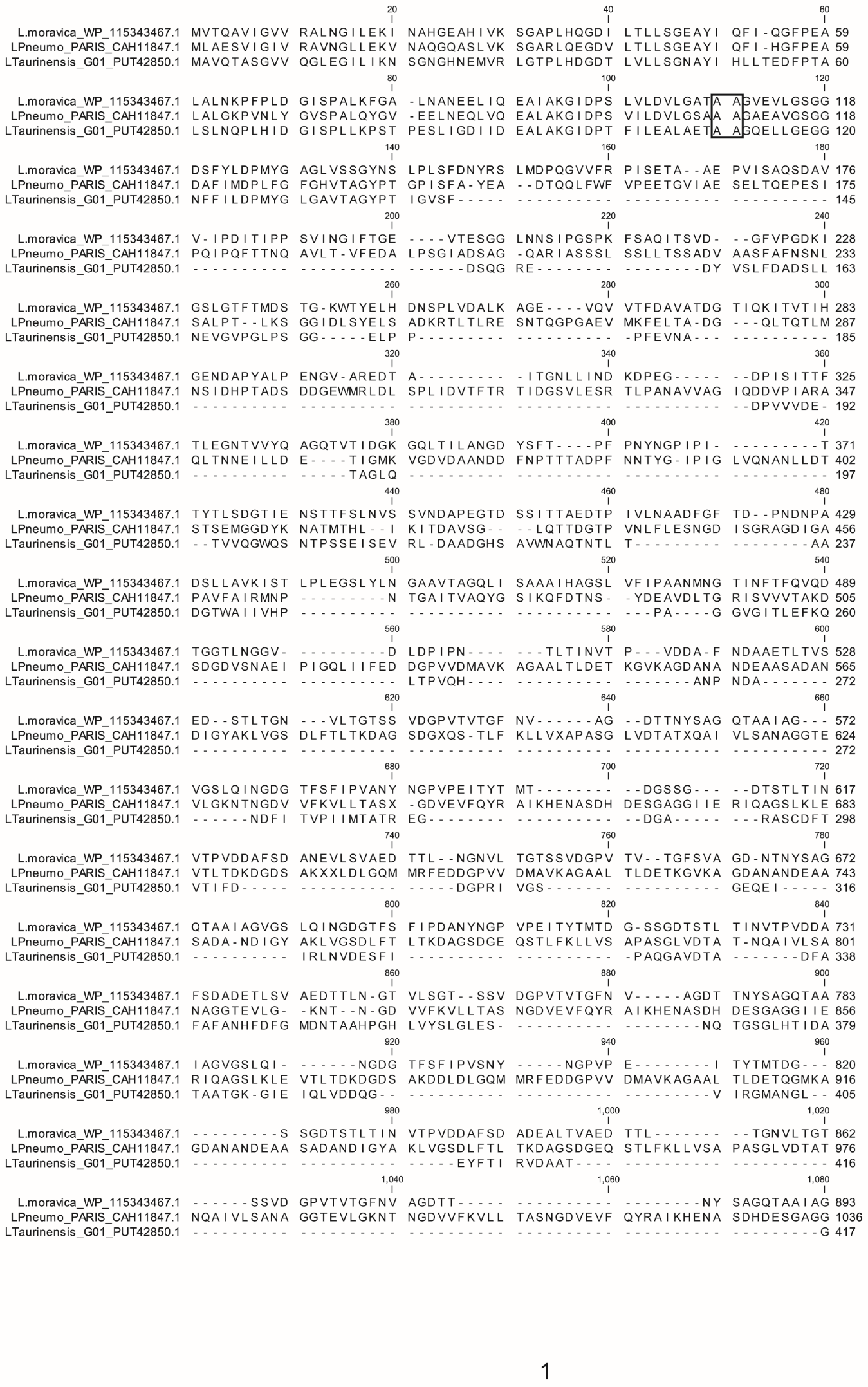
**

**Figure D.** Partial alignment of *L. pneumophila* Paris, *L. taurinensis* Genessee01, and *L. moravica* RtxA. The black box encloses the sequences of the retention module [1, 2].


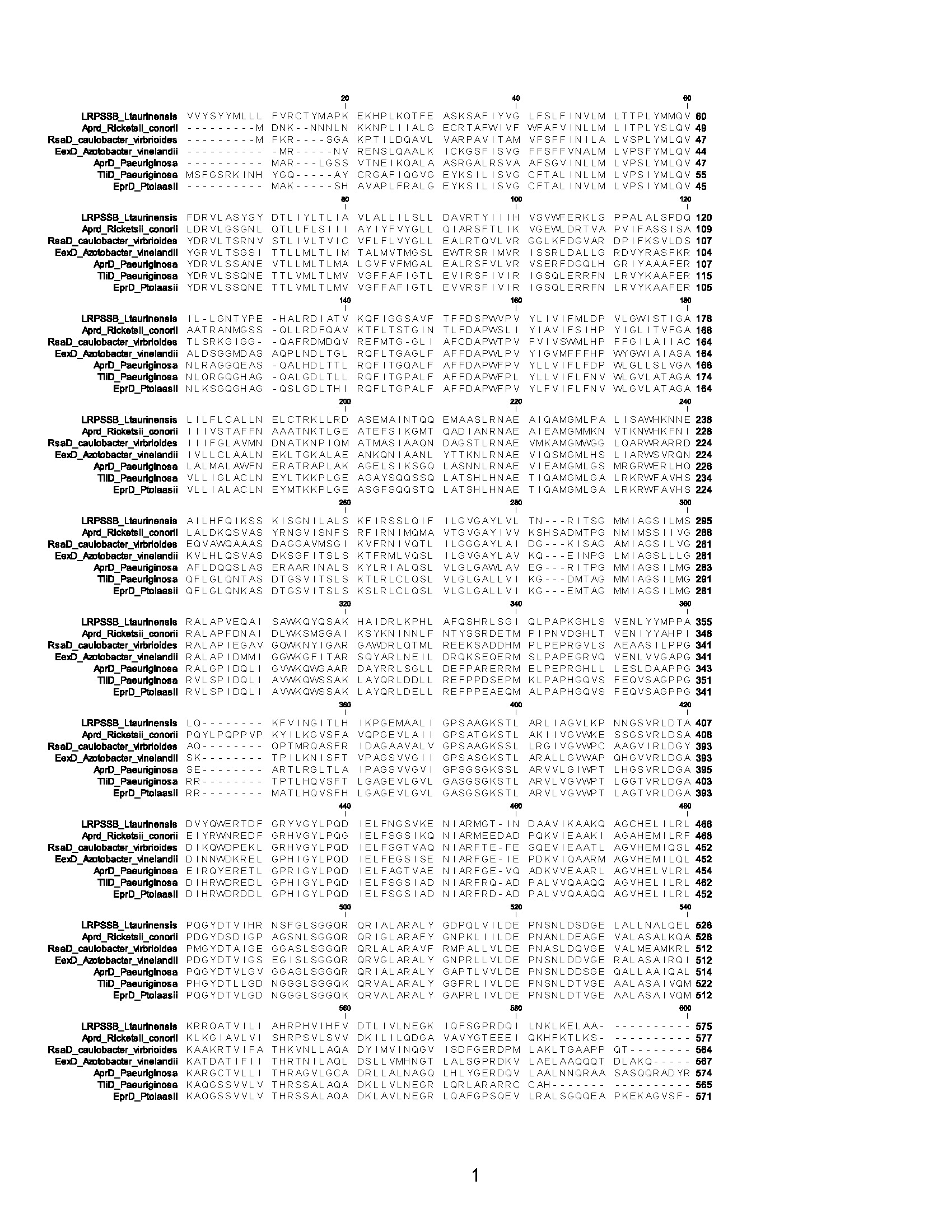


**Figure E.** Alignment of T1SS ABC transporters which do not possess N-terminal functional domains, including LrpssB discovered in *L. taurinensis.*


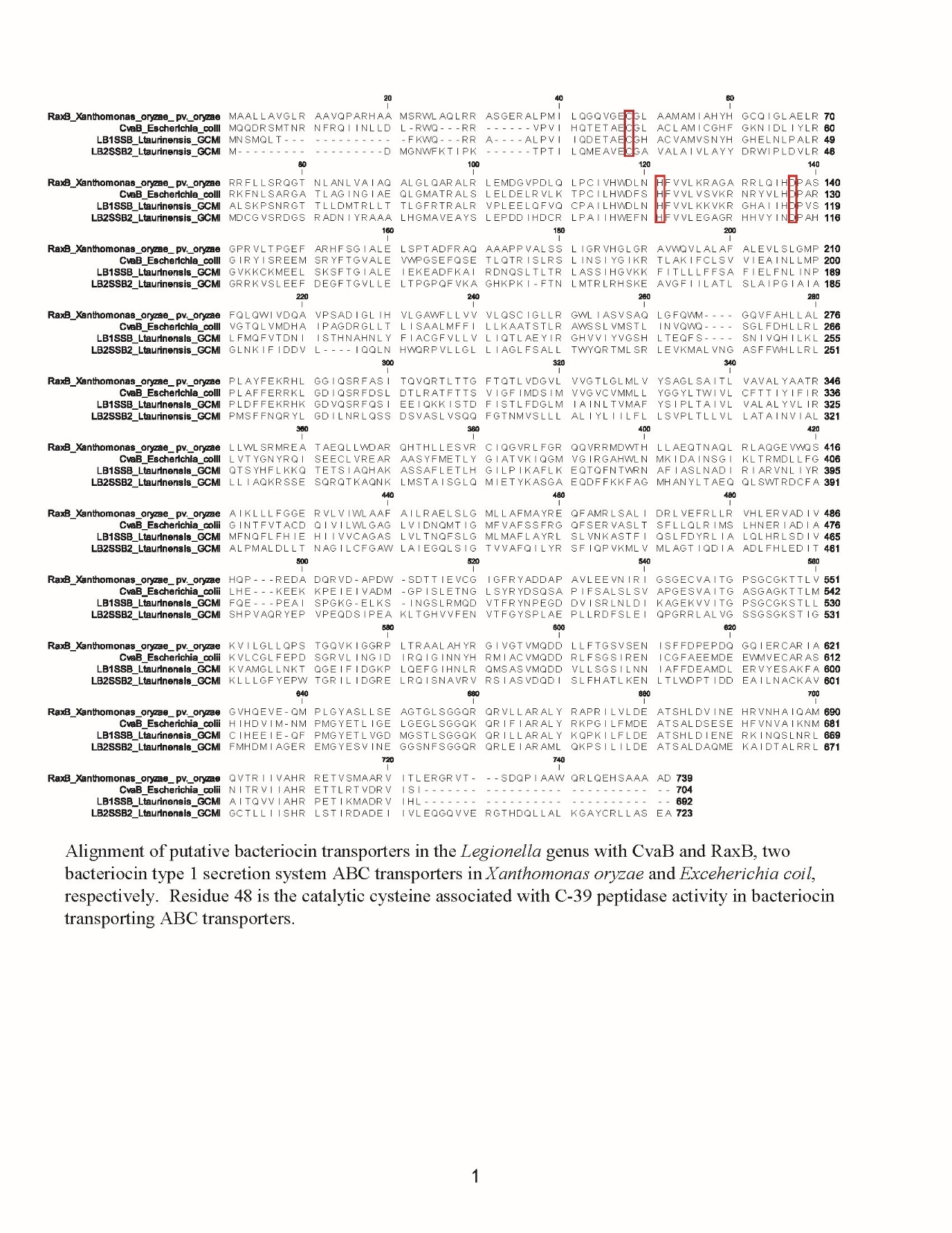


**Figure F.** Alignment of C-39 peptidase-type T1SS ABC transporters including the ABC transporters of two novel T1SSs found throughout the *Legionella* genus. Both putative bacteriocin transporter ABC transporters contain C-39 peptidase motifs (red boxes) consistent with previously characterized system.

| **Table B.** NCBI Sequence IDs and BLAST results of *Legionella* T1SS components. All percent identities are adjusted for incomplete query covers, i.e. % query cover multiplied by % identity. | | | | | | |
| --- | --- | --- | --- | --- | --- | --- |
| **Query Protein** | **Query Strain** | **Query Sequence ID** | **Subject Sequence ID** | **Subject Strain** | **% Identity** | **Suggested Name** |
| LssB | Legionella pneumophila Philadelphia 1 ATCC 33512 | CAD90959.1 | [KTD81364.1](https://www.ncbi.nlm.nih.gov/protein/KTD81364.1?report=genbank&log$=protalign&blast_rank=3&RID=82HV69M7014) | *Legionella worsliensis* ATCC 49508 | 78 | LssB |
| LssB | Legionella pneumophila Philadelphia 1 ATCC 33512 | CAD90959.1 | [STX62775.1](https://www.ncbi.nlm.nih.gov/protein/STX62775.1?report=genbank&log$=protalign&blast_rank=1&RID=82FDAA81014) | *Legionella moravica* NCTC12239 | 81 | LssB |
| LssB | Legionella pneumophila Philadelphia 1 ATCC 33512 | CAD90959.1 | [WP_058473318.1](https://www.ncbi.nlm.nih.gov/protein/WP_058473318.1?report=genbank&log$=protalign&blast_rank=2&RID=82HV69M7014) | *Legionella quateirensis* NCTC12376 | 81 | LssB |
| LssB | Legionella pneumophila Philadelphia 1 ATCC 33512 | CAD90959.1 | [WP_058481714.1](https://www.ncbi.nlm.nih.gov/protein/WP_058481714.1?report=genbank&log$=protalign&blast_rank=6&RID=82HV69M7014) | *Legionella waltersii* NCTC13017 | 75.45 | LssB |
| LssB | Legionella pneumophila Philadelphia 1 ATCC 33512 | CAD90959.1 | [WP_035886095.1](https://www.ncbi.nlm.nih.gov/protein/WP_035886095.1?report=genbank&log$=protalign&blast_rank=1&RID=82HV69M7014) | *Legionella norrlandica* LEGN | 87 | LssB |
| LssB | Legionella pneumophila Philadelphia 1 ATCC 33512 | CAD90959.1 | [WP_058497724.1](https://www.ncbi.nlm.nih.gov/protein/WP_058497724.1?report=genbank&log$=protalign&blast_rank=12&RID=82HV69M7014) | *Legionella gratiana* NCTC12388 | 62.3 | LssB |
| LssB | Legionella pneumophila Philadelphia 1 ATCC 33512 | CAD90959.1 | [WP_058514852.1](https://www.ncbi.nlm.nih.gov/protein/WP_058514852.1?report=genbank&log$=protalign&blast_rank=15&RID=82HV69M7014) | *Legionella santicrusis* SC-63-C7 | 61 | LssB |
| LssB | Legionella pneumophila Philadelphia 1 ATCC 33512 | CAD90959.1 | [ARM32591.1](https://www.ncbi.nlm.nih.gov/protein/ARM32591.1?report=genbank&log$=protalign&blast_rank=1&RID=82NSSEVM014) | *Legionella longbeachae* F1157CHC | partial* | LssB |
| LssB | Legionella pneumophila Philadelphia 1 ATCC 33512 | CAD90959.1 | [RZV21064.1](https://www.ncbi.nlm.nih.gov/protein/RZV21064.1?report=genbank&log$=protalign&blast_rank=2&RID=82NSSEVM014) | *Legionella longbeachae* FDAARGOS201 | partial* | LssB |
| LssB | Legionella pneumophila Philadelphia 1 ATCC 33512 | CAD90959.1 | [WP_064276704.1](https://www.ncbi.nlm.nih.gov/protein/WP_064276704.1?report=genbank&log$=protalign&blast_rank=19&RID=82HV69M7014) | *Legionella gormanii* NCTC11401 | 43 | LapB-type ABC transporter |
| LssB | Legionella pneumophila Philadelphia 1 ATCC 33512 | CAD90959.1 | [WP_058440475.1](https://www.ncbi.nlm.nih.gov/protein/WP_058440475.1?report=genbank&log$=protalign&blast_rank=20&RID=82HV69M7014) | *Legionella brunensis ATCC 43878* | 41.58 | LapB-type ABC transporter |
| LssB | Legionella pneumophila Philadelphia 1 ATCC 33512 | CAD90959.1 | [WP_045106012.1](https://www.ncbi.nlm.nih.gov/protein/WP_045106012.1?report=genbank&log$=protalign&blast_rank=13&RID=82HV69M7014) | *Legionella hackeliae NCTC11979* | 64.62 | LssB |
| LssB | Legionella pneumophila Philadelphia 1 ATCC 33512 | CAD90959.1 | [WP_028372976.1](https://www.ncbi.nlm.nih.gov/protein/WP_028372976.1?report=genbank&log$=protalign&blast_rank=16&RID=82HV69M7014) | *Legionella lansingensis* NCTC12830 | 64.35 | LssB |
| LssB | Legionella pneumophila Philadelphia 1 ATCC 33512 | CAD90959.1 | [WP_115175174.1](https://www.ncbi.nlm.nih.gov/protein/WP_115175174.1?report=genbank&log$=protalign&blast_rank=8&RID=82HV69M7014) | *Legionella feeleii* NCTC11978 | 67 | LssB |
| LssB | Legionella pneumophila Philadelphia 1 ATCC 33512 | CAD90959.1 | [WP_126338036.1](https://www.ncbi.nlm.nih.gov/protein/WP_126338036.1?report=genbank&log$=protalign&blast_rank=10&RID=82HV69M7014) | *Legionella spiritensis* NCTC12082 | 66.3 | LssB |
| LssB | Legionella pneumophila Philadelphia 1 ATCC 33512 | CAD90959.1 | [WP_058526275.1](https://www.ncbi.nlm.nih.gov/protein/WP_058526275.1?report=genbank&log$=protalign&blast_rank=1&RID=82KZSV0C014) | *Legionella erythra* SE-32A-C8 | 65 | LssB |
| LssB | Legionella pneumophila Philadelphia 1 ATCC 33512 | CAD90959.1 | [WP_058530189.1](https://www.ncbi.nlm.nih.gov/protein/WP_058530189.1?report=genbank&log$=protalign&blast_rank=2&RID=82KZSV0C014) | *Legionella rubrilucens* WA-270A-C2 | 66 | LssB |
| LssB | Legionella pneumophila Philadelphia 1 ATCC 33512 | CAD90959.1 | [WP_108291611.1](https://www.ncbi.nlm.nih.gov/protein/WP_108291611.1?report=genbank&log$=protalign&blast_rank=14&RID=82HV69M7014) | *Legionella taurinensis* GCMI-01 | 65 | LssB |
| LssB | Legionella pneumophila Philadelphia 1 ATCC 33512 | CAD90959.1 | [WP_058524103.1](https://www.ncbi.nlm.nih.gov/protein/WP_058524103.1?report=genbank&log$=protalign&blast_rank=18&RID=82HV69M7014) | *Legionella birminghamensis* NCTC12437 | 65.46 | LssB |
| LssB | Legionella pneumophila Philadelphia 1 ATCC 33512 | CAD90959.1 | [WP_058508324.1](https://www.ncbi.nlm.nih.gov/protein/WP_058508324.1?report=genbank&log$=protalign&blast_rank=17&RID=82HV69M7014) | *Legionella quinlivani* NCTC12433 | 64.9 | LssB |
| LssB | Legionella pneumophila Philadelphia 1 ATCC 33512 | CAD90959.1 | [WP_058502292.1](https://www.ncbi.nlm.nih.gov/protein/WP_058502292.1?report=genbank&log$=protalign&blast_rank=7&RID=82HV69M7014) | *Legionella israelensis* NCTC12010 | 68.38 | LssB |
| LssD | Legionella pneumophila Philadelphia 1 ATCC 33512 | CAD90958.1 | [WP_018577805.1](https://www.ncbi.nlm.nih.gov/protein/WP_018577805.1?report=genbank&log$=protalign&blast_rank=4&RID=8079JU23014) | *Legionella shakespearei* DSM23087 | 79.37 | LssD |
| LssD | Legionella pneumophila Philadelphia 1 ATCC 33512 | CAD90958.1 | [WP_058492478.1](https://www.ncbi.nlm.nih.gov/protein/WP_058492478.1?report=genbank&log$=protalign&blast_rank=2&RID=8079JU23014) | *Legionella worsliensis* NCTC12377 | 80.16 | LssD |
| LssD | Legionella pneumophila Philadelphia 1 ATCC 33512 | CAD90958.1 | [WP_028384637.1](https://www.ncbi.nlm.nih.gov/protein/WP_028384637.1?report=genbank&log$=protalign&blast_rank=1&RID=82FC6STJ014) | *Legionella moravica* NCTC12239 | 84 | LssD |
| LssD | Legionella pneumophila Philadelphia 1 ATCC 33512 | CAD90958.1 | [WP_058473319.1](https://www.ncbi.nlm.nih.gov/protein/WP_058473319.1?report=genbank&log$=protalign&blast_rank=1&RID=8079JU23014) | *Legionella quateirensis* NCTC12376 | 83.86 | LssD |
| LssD | Legionella pneumophila Philadelphia 1 ATCC 33512 | CAD90958.1 | [WP_058481713.1](https://www.ncbi.nlm.nih.gov/protein/WP_058481713.1?report=genbank&log$=protalign&blast_rank=3&RID=8079JU23014) | *Legionella waltersii* NCTC13017 | 81 | LssD |
| LssD | Legionella pneumophila Philadelphia 1 ATCC 33512 | CAD90958.1 | [WP_058497725.1](https://www.ncbi.nlm.nih.gov/protein/WP_058497725.1?report=genbank&log$=protalign&blast_rank=15&RID=8079JU23014) | *Legionella gratiana* NCTC12388 | 61 | LssD |
| LssD | Legionella pneumophila Philadelphia 1 ATCC 33512 | CAD90958.1 | [WP_058514851.1](https://www.ncbi.nlm.nih.gov/protein/WP_058514851.1?report=genbank&log$=protalign&blast_rank=14&RID=8079JU23014) | *Legionella santicrusis* SC-63-C7 | 62 | LssD |
| LssD | Legionella pneumophila Philadelphia 1 ATCC 33512 | CAD90958.1 | [ARM32590.1](https://www.ncbi.nlm.nih.gov/protein/ARM32590.1?report=genbank&log$=protalign&blast_rank=1&RID=82NJP36N015) | *Legionella longbeachae* F1157CHC | partial* | LssD |
| LssD | Legionella pneumophila Philadelphia 1 ATCC 33512 | CAD90958.1 | [RZV21065.1](https://www.ncbi.nlm.nih.gov/protein/RZV21065.1?report=genbank&log$=protalign&blast_rank=1&RID=82NJP36N015) | *Legionella longbeachae* FDAARGOS201 | partial* | LssD |
| LssD | Legionella pneumophila Philadelphia 1 ATCC 33512 | CAD90958.1 | WP_058440476.1 | *Legionella brunensis ATCC 43878* | 48 | LapD-type MFP |
| LssD | Legionella pneumophila Philadelphia 1 ATCC 33512 | CAD90958.1 | [WP_045106011.1](https://www.ncbi.nlm.nih.gov/protein/WP_045106011.1?report=genbank&log$=protalign&blast_rank=5&RID=8079JU23014) | *Legionella hackeliae NCTC11979 NCTC11979* | 68.34 | LssD |
| LssD | Legionella pneumophila Philadelphia 1 ATCC 33512 | CAD90958.1 | [WP_028372975.1](https://www.ncbi.nlm.nih.gov/protein/WP_028372975.1?report=genbank&log$=protalign&blast_rank=10&RID=8079JU23014) | *Legionella lansingensis* NCTC12830 | 82 | LssD |
| LssD | Legionella pneumophila Philadelphia 1 ATCC 33512 | CAD90958.1 | [WP_115175175.1](https://www.ncbi.nlm.nih.gov/protein/WP_115175175.1?report=genbank&log$=protalign&blast_rank=2&RID=82H9EVZ4015) | *Legionella feeleii* NCTC11978 | 67.3 | LssD |
| LssD | Legionella pneumophila Philadelphia 1 ATCC 33512 | CAD90958.1 | [WP_126338038.1](https://www.ncbi.nlm.nih.gov/protein/WP_126338038.1?report=genbank&log$=protalign&blast_rank=12&RID=8079JU23014) | *Legionella spiritensis* NCTC12082 | 64.29 | LssD |
| LssD | Legionella pneumophila Philadelphia 1 ATCC 33512 | CAD90958.1 | [WP_058526274.1](https://www.ncbi.nlm.nih.gov/protein/WP_058526274.1?report=genbank&log$=protalign&blast_rank=2&RID=82NFF8R4014) | *Legionella erythra* SE-32A-C8 | 64 | LssD |
| LssD | Legionella pneumophila Philadelphia 1 ATCC 33512 | CAD90958.1 | [WP_058530190.1](https://www.ncbi.nlm.nih.gov/protein/WP_058530190.1?report=genbank&log$=protalign&blast_rank=1&RID=82NFF8R4014) | *Legionella rubrilucens* WA-270A-C2 | 64 | LssD |
| LssD | Legionella pneumophila Philadelphia 1 ATCC 33512 | CAD90958.1 | [WP_108291613.1](https://www.ncbi.nlm.nih.gov/protein/WP_108291613.1?report=genbank&log$=protalign&blast_rank=11&RID=8079JU23014) | *Legionella taurinensis* GCMI-01 | 63 | LssD |
| LssD | Legionella pneumophila Philadelphia 1 ATCC 33512 | CAD90958.1 | [WP_058524102.1](https://www.ncbi.nlm.nih.gov/protein/WP_058524102.1?report=genbank&log$=protalign&blast_rank=8&RID=8079JU23014) | *Legionella birminghamensis* NCTC12437 | 67.4 | LssD |
| LssD | Legionella pneumophila Philadelphia 1 ATCC 33512 | CAD90958.1 | [WP_058508325.1](https://www.ncbi.nlm.nih.gov/protein/WP_058508325.1?report=genbank&log$=protalign&blast_rank=9&RID=8079JU23014) | *Legionella quinlivani* NCTC12433 | 67 | LssD |
| LssD | Legionella pneumophila Philadelphia 1 ATCC 33512 | CAD90958.1 | [WP_058502291.1](https://www.ncbi.nlm.nih.gov/protein/WP_058502291.1?report=genbank&log$=protalign&blast_rank=6&RID=8079JU23014) | *Legionella israelensis* NCTC12010 | 68 | LssD |
| LrpssB | Legionella taurinensis GCMI-01 | PUT43961.1 | [WP_026253944.1](https://www.ncbi.nlm.nih.gov/protein/WP_026253944.1?report=genbank&log$=protalign&blast_rank=11&RID=82RKAF0D015) | *Legionella shakespearei* DSM23087 | 58.51 | LrpssB |
| LrpssB | Legionella taurinensis GCMI-01 | PUT43961.1 | [WP_051546306.1](https://www.ncbi.nlm.nih.gov/protein/WP_051546306.1?report=genbank&log$=protalign&blast_rank=7&RID=82RKAF0D015) | *Legionella feeleii* NCTC11978 | 70 | LrpssB |
| LrpssB | Legionella taurinensis GCMI-01 | PUT43961.1 | [WP_126337954.1](https://www.ncbi.nlm.nih.gov/protein/WP_126337954.1?report=genbank&log$=protalign&blast_rank=5&RID=82RKAF0D015) | *Legionella spiritensis* NCTC12082 | 68 | LrpssB |
| LrpssB | Legionella taurinensis GCMI-01 | PUT43961.1 | [WP_065235700.1](https://www.ncbi.nlm.nih.gov/protein/WP_065235700.1?report=genbank&log$=protalign&blast_rank=1&RID=82SC8D2U015) | *Legionella rubrilucens* WA-270A-C2 | 97 | LrpssB |
| LrpssB | Legionella taurinensis GCMI-01 | PUT43961.1 | [WP_051399027.1](https://www.ncbi.nlm.nih.gov/protein/WP_051399027.1?report=genbank&log$=protalign&blast_rank=13&RID=82RKAF0D015) | *Legionella oakridgensis* RV-2-2007 | 46 | LrpssB |
| LrpssB | Legionella taurinensis GCMI-01 | PUT43961.1 | [WP_094091207.1](https://www.ncbi.nlm.nih.gov/protein/WP_094091207.1?report=genbank&log$=protalign&blast_rank=3&RID=82RKAF0D015) | *Legionella clemsonensis CDC-D5610* | 70 | LrpssB |
| LrpssB | Legionella taurinensis GCMI-01 | PUT43961.1 | [WP_058448978.1](https://www.ncbi.nlm.nih.gov/protein/WP_058448978.1?report=genbank&log$=protalign&blast_rank=4&RID=82RKAF0D015) | *Legionella jamestowniensis DSM 19215* | 70 | LrpssB |
| LrpssB | Legionella taurinensis GCMI-01 | PUT43961.1 | WP_115678688.1 | *Legionella hackeliae NCTC11979* | 69 | LrpssB |
| LrpssB | Legionella taurinensis GCMI-01 | PUT43961.1 | [WP_058479565.1](https://www.ncbi.nlm.nih.gov/protein/WP_058479565.1?report=genbank&log$=protalign&blast_rank=1&RID=82TGAB9F015) | *Legionella waltersii* NCTC13017 | 53 | LrpssB |
| LrpssD | Legionella taurinensis GCMI-01 | PUT43962.1 | [WP_018577319.1](https://www.ncbi.nlm.nih.gov/protein/WP_018577319.1?report=genbank&log$=protalign&blast_rank=13&RID=82SZ8BSN015) | *Legionella shakespearei* DSM23087 | 53 | LrpssD |
| LrpssD | Legionella taurinensis GCMI-01 | PUT43962.1 | [WP_051546303.1](https://www.ncbi.nlm.nih.gov/protein/WP_051546303.1?report=genbank&log$=protalign&blast_rank=10&RID=82SZ8BSN015) | *Legionella lansingensis* NCTC12830 | 69 | LrpssD |
| LrpssD | Legionella taurinensis GCMI-01 | PUT43962.1 | [KTD64080.1](https://www.ncbi.nlm.nih.gov/protein/KTD64080.1?report=genbank&log$=protalign&blast_rank=3&RID=82SZ8BSN015) | *Legionella spiritensis* NCTC12082 | 72 | LrpssD |
| LrpssD | Legionella taurinensis GCMI-01 | PUT43962.1 | [WP_058530640.1](https://www.ncbi.nlm.nih.gov/protein/WP_058530640.1?report=genbank&log$=protalign&blast_rank=2&RID=82SZ8BSN015) | *Legionella rubrilucens* WA-270A-C2 | 97.72 | LrpssD |
| LrpssD | Legionella taurinensis GCMI-01 | PUT43962.1 | [WP_051399026.1](https://www.ncbi.nlm.nih.gov/protein/WP_051399026.1?report=genbank&log$=protalign&blast_rank=16&RID=82SZ8BSN015) | *Legionella oakridgensis RV-2-2007* | 44 | LrpssD |
| LrpssD | Legionella taurinensis GCMI-01 | PUT43962.1 | [WP_094091208.1](https://www.ncbi.nlm.nih.gov/protein/WP_094091208.1?report=genbank&log$=protalign&blast_rank=8&RID=82SZ8BSN015) | *Legionella clemsonensis CDC-D5610* | 70 | LrpssD |
| LrpssD | Legionella taurinensis GCMI-01 | PUT43962.1 | [WP_082651575.1](https://www.ncbi.nlm.nih.gov/protein/WP_082651575.1?report=genbank&log$=protalign&blast_rank=6&RID=82SZ8BSN015) | *Legionella jamestowniensis DSM 19215* | 71 | LrpssD |
| LrpssD | Legionella taurinensis GCMI-01 | PUT43962.1 | [WP_082060296.1](https://www.ncbi.nlm.nih.gov/protein/WP_082060296.1?report=genbank&log$=protalign&blast_rank=11&RID=82SZ8BSN015) | *Legionella hackeliae NCTC11979* | 69 | LrpssD |
| LrpssD | Legionella taurinensis GCMI-01 | PUT43962.1 | [WP_058479564.1](https://www.ncbi.nlm.nih.gov/protein/WP_058479564.1?report=genbank&log$=protalign&blast_rank=14&RID=82SZ8BSN015) | *Legionella waltersii NCTC13017* | 58 | LrpssD |
| Lb1ssB | Legionella taurinensis GCMI-01 | PUT42244.1 | [WP_106184359.1](https://www.ncbi.nlm.nih.gov/protein/WP_106184359.1?report=genbank&log$=protalign&blast_rank=21&RID=82V1K4SD015) | *Legionella pneumophila* | 53 | Lb1ssB |
| Lb1ssB | Legionella taurinensis GCMI-01 | PUT42244.1 | WP_012979428.1 | *Legionella longbeachae NSW150* | 54 | Lb1ssB |
| Lb1ssB | Legionella taurinensis GCMI-01 | PUT42244.1 | [KTC86663.1](https://www.ncbi.nlm.nih.gov/protein/KTC86663.1?report=genbank&log$=protalign&blast_rank=22&RID=82V1K4SD015) | *Legionella brunensis ATCC 43878* | 53 | Lb1ssB |
| Lb1ssB | Legionella taurinensis GCMI-01 | PUT42244.1 | [WP_045107270.1](https://www.ncbi.nlm.nih.gov/protein/WP_045107270.1?report=genbank&log$=protalign&blast_rank=6&RID=82V1K4SD015) | *Legionella hackeliae NCTC11979* | 61 | Lb1ssB |
| Lb1ssB | Legionella taurinensis GCMI-01 | PUT42244.1 | [WP_094089766.1](https://www.ncbi.nlm.nih.gov/protein/WP_094089766.1?report=genbank&log$=protalign&blast_rank=12&RID=82V1K4SD015) | *Legionella clemsonensis CDC-D5610* | 60 | Lb1ssB |
| Lb1ssB | Legionella taurinensis GCMI-01 | PUT42244.1 | [WP_058449780.1](https://www.ncbi.nlm.nih.gov/protein/WP_058449780.1?report=genbank&log$=protalign&blast_rank=11&RID=82V1K4SD015) | *Legionella jamestowniensis DSM 19215* | 59 | Lb1ssB |
| Lb1ssB | Legionella taurinensis GCMI-01 | PUT42244.1 | [WP_081778091.1](https://www.ncbi.nlm.nih.gov/protein/WP_081778091.1?report=genbank&log$=protalign&blast_rank=9&RID=82V1K4SD015) | *Legionella lansingensis* NCTC12830 | 60 | Lb1ssB |
| Lb1ssB | Legionella taurinensis GCMI-01 | PUT42244.1 | [WP_058470006.1](https://www.ncbi.nlm.nih.gov/protein/WP_058470006.1?report=genbank&log$=protalign&blast_rank=8&RID=82V1K4SD015) | *Legionella jordanis NCTC11533* | 58 | Lb1ssB |
| Lb1ssB | Legionella taurinensis GCMI-01 | PUT42244.1 | [WP_058446794.1](https://www.ncbi.nlm.nih.gov/protein/WP_058446794.1?report=genbank&log$=protalign&blast_rank=17&RID=82V1K4SD015) | *Legionella feeleii* NCTC11978 | 59 | Lb1ssB |
| Lb1ssB | Legionella taurinensis GCMI-01 | PUT42244.1 | [WP_084273757.1](https://www.ncbi.nlm.nih.gov/protein/WP_084273757.1?report=genbank&log$=protalign&blast_rank=16&RID=82V1K4SD015) | *Legionella fairfieldensis ATCC 49588* | 59 | Lb1ssB |
| Lb1ssB | Legionella taurinensis GCMI-01 | PUT42244.1 | [WP_058505330.1](https://www.ncbi.nlm.nih.gov/protein/WP_058505330.1?report=genbank&log$=protalign&blast_rank=25&RID=82V1K4SD015) | *Legionella nautarum ATCC 49506* | 58 | Lb1ssB |
| Lb1ssB | Legionella taurinensis GCMI-01 | PUT42244.1 | [WP_126338215.1](https://www.ncbi.nlm.nih.gov/protein/WP_126338215.1?report=genbank&log$=protalign&blast_rank=15&RID=82V1K4SD015) | *Legionella spiritensis NCTC12082* | 58 | Lb1ssB |
| Lb1ssB | Legionella taurinensis GCMI-01 | PUT42244.1 | [WP_058531658.1](https://www.ncbi.nlm.nih.gov/protein/WP_058531658.1?report=genbank&log$=protalign&blast_rank=5&RID=82V1K4SD015) | *Legionella rubrilucens WA-270A-C2* | 92 | Lb1ssB |
| Lb1ssB | Legionella taurinensis GCMI-01 | PUT42244.1 | [KTC64807.1](https://www.ncbi.nlm.nih.gov/protein/KTC64807.1?report=genbank&log$=protalign&blast_rank=19&RID=82V1K4SD015) | *Legionella adelaidensis 1762-AUS-E* | 57 | Lb1ssB |
| Lb1ssB | Legionella taurinensis GCMI-01 | PUT42244.1 | [WP_035893151.1](https://www.ncbi.nlm.nih.gov/protein/WP_035893151.1?report=genbank&log$=protalign&blast_rank=7&RID=82V1K4SD015) | *Legionella oakridgensis RV-2-2007* | 40 | Lb1ssB |
| Lb1ssD | Legionella taurinensis GCMI-01 | [PUT42245.1](https://www.ncbi.nlm.nih.gov/protein/PUT42245.1?report=genbank&log$=protalign&blast_rank=1&RID=82UXXCXR015) | WP_012979429 | *Legionella longbeachae* NSW150 | 35 | Lb1ssD |
| Lb1ssD | Legionella taurinensis GCMI-01 | [PUT42245.1](https://www.ncbi.nlm.nih.gov/protein/PUT42245.1?report=genbank&log$=protalign&blast_rank=1&RID=82UXXCXR015) | [WP_058440694.1](https://www.ncbi.nlm.nih.gov/protein/WP_058440694.1?report=genbank&log$=protalign&blast_rank=17&RID=87ZXE8H7015) | *Legionella brunensis ATCC 43878* | 35 | Lb1ssD |
| Lb1ssD | Legionella taurinensis GCMI-01 | [PUT42245.1](https://www.ncbi.nlm.nih.gov/protein/PUT42245.1?report=genbank&log$=protalign&blast_rank=1&RID=82UXXCXR015) | [WP_082060375.1](https://www.ncbi.nlm.nih.gov/protein/WP_082060375.1?report=genbank&log$=protalign&blast_rank=14&RID=87ZXE8H7015) | *Legionella hackeliae NCTC11979* | 38 | Lb1ssD |
| Lb1ssD | Legionella taurinensis GCMI-01 | [PUT42245.1](https://www.ncbi.nlm.nih.gov/protein/PUT42245.1?report=genbank&log$=protalign&blast_rank=1&RID=82UXXCXR015) | [ASQ44611.1](https://www.ncbi.nlm.nih.gov/protein/ASQ44611.1?report=genbank&log$=protalign&blast_rank=12&RID=87ZXE8H7015) | *Legionella clemsonensis CDC-D5610* | 38.72 | Lb1ssD |
| Lb1ssD | Legionella taurinensis GCMI-01 | [PUT42245.1](https://www.ncbi.nlm.nih.gov/protein/PUT42245.1?report=genbank&log$=protalign&blast_rank=1&RID=82UXXCXR015) | [WP_058449781.1](https://www.ncbi.nlm.nih.gov/protein/WP_058449781.1?report=genbank&log$=protalign&blast_rank=9&RID=87ZXE8H7015) | *Legionella jamestowniensis DSM 19215* | 38.41 | Lb1ssD |
| Lb1ssD | Legionella taurinensis GCMI-01 | [PUT42245.1](https://www.ncbi.nlm.nih.gov/protein/PUT42245.1?report=genbank&log$=protalign&blast_rank=1&RID=82UXXCXR015) | [WP_028373626.1](https://www.ncbi.nlm.nih.gov/protein/WP_028373626.1?report=genbank&log$=protalign&blast_rank=10&RID=87ZXE8H7015) | *Legionella lansingensis* NCTC12830 | 41 | Lb1ssD |
| Lb1ssD | Legionella taurinensis GCMI-01 | [PUT42245.1](https://www.ncbi.nlm.nih.gov/protein/PUT42245.1?report=genbank&log$=protalign&blast_rank=1&RID=82UXXCXR015) | [WP_058470007.1](https://www.ncbi.nlm.nih.gov/protein/WP_058470007.1?report=genbank&log$=protalign&blast_rank=3&RID=82W8Z58A014) | *Legionella jordanis* NCTC11533 | 42 | Lb1ssD |
| Lb1ssD | Legionella taurinensis GCMI-01 | [PUT42245.1](https://www.ncbi.nlm.nih.gov/protein/PUT42245.1?report=genbank&log$=protalign&blast_rank=1&RID=82UXXCXR015) | [WP_058446796.1](https://www.ncbi.nlm.nih.gov/protein/WP_058446796.1?report=genbank&log$=protalign&blast_rank=4&RID=87ZXE8H7015) | *Legionella feeleii* NCTC11978 | 43 | Lb1ssD |
| Lb1ssD | Legionella taurinensis GCMI-01 | [PUT42245.1](https://www.ncbi.nlm.nih.gov/protein/PUT42245.1?report=genbank&log$=protalign&blast_rank=1&RID=82UXXCXR015) | [WP_035918432.1](https://www.ncbi.nlm.nih.gov/protein/WP_035918432.1?report=genbank&log$=protalign&blast_rank=11&RID=87ZXE8H7015) | *Legionella fairfieldensis ATCC 49588* | 40.24 | Lb1ssD |
| Lb1ssD | Legionella taurinensis GCMI-01 | [PUT42245.1](https://www.ncbi.nlm.nih.gov/protein/PUT42245.1?report=genbank&log$=protalign&blast_rank=1&RID=82UXXCXR015) | [WP_058505331.1](https://www.ncbi.nlm.nih.gov/protein/WP_058505331.1?report=genbank&log$=protalign&blast_rank=19&RID=87ZXE8H7015) | *Legionella nautarum ATCC 49506* | 35 | Lb1ssD |
| Lb1ssD | Legionella taurinensis GCMI-01 | [PUT42245.1](https://www.ncbi.nlm.nih.gov/protein/PUT42245.1?report=genbank&log$=protalign&blast_rank=1&RID=82UXXCXR015) | [WP_058484230.1](https://www.ncbi.nlm.nih.gov/protein/WP_058484230.1?report=genbank&log$=protalign&blast_rank=7&RID=87ZXE8H7015) | *Legionella spiritensis* NCTC12082 | 38.25 | Lb1ssD |
| Lb1ssD | Legionella taurinensis GCMI-01 | [PUT42245.1](https://www.ncbi.nlm.nih.gov/protein/PUT42245.1?report=genbank&log$=protalign&blast_rank=1&RID=82UXXCXR015) | [WP_058531657.1](https://www.ncbi.nlm.nih.gov/protein/WP_058531657.1?report=genbank&log$=protalign&blast_rank=2&RID=87ZXE8H7015) | *Legionella rubrilucens* WA-270A-C2 | 88.25 | Lb1ssD |
| Lb1ssD | Legionella taurinensis GCMI-01 | [PUT42245.1](https://www.ncbi.nlm.nih.gov/protein/PUT42245.1?report=genbank&log$=protalign&blast_rank=1&RID=82UXXCXR015) | [WP_058463151.1](https://www.ncbi.nlm.nih.gov/protein/WP_058463151.1?report=genbank&log$=protalign&blast_rank=18&RID=87ZXE8H7015) | *Legionella adelaidensis 1762-AUS-E* | 37 | Lb1ssD |
| Lb1ssD | Legionella taurinensis GCMI-01 | [PUT42245.1](https://www.ncbi.nlm.nih.gov/protein/PUT42245.1?report=genbank&log$=protalign&blast_rank=1&RID=82UXXCXR015) | [WP_035893149.1](https://www.ncbi.nlm.nih.gov/protein/WP_035893149.1?report=genbank&log$=protalign&blast_rank=6&RID=87ZXE8H7015) | *Legionella oakridgensis RV-2-2007* | 39.76 | Lb1ssD |
| Lb2ssB | Legionella taurinensis GCMI-01 | PUT41642.1 | [WP_058473476.1](https://www.ncbi.nlm.nih.gov/protein/WP_058473476.1?report=genbank&log$=protalign&blast_rank=14&RID=881ZAR9G015) | *Legionella quateirensis NCTC12376* | 44 | Lb2ssB |
| Lb2ssB | Legionella taurinensis GCMI-01 | PUT41642.1 | [WP_045094812.1](https://www.ncbi.nlm.nih.gov/protein/WP_045094812.1?report=genbank&log$=protalign&blast_rank=11&RID=881ZAR9G015) | *Legionella fallonii LLAP-10* | 71 | Lb2ssB |
| Lb2ssB | Legionella taurinensis GCMI-01 | PUT41642.1 | [WP_058481063.1](https://www.ncbi.nlm.nih.gov/protein/WP_058481063.1?report=genbank&log$=protalign&blast_rank=13&RID=881ZAR9G015) | *Legionella waltersii NCTC13017* | 43 | NHLP-type T1SS ABC transporter |
| Lb2ssB | Legionella taurinensis GCMI-01 | PUT41642.1 | [WP_058498779.1](https://www.ncbi.nlm.nih.gov/protein/WP_058498779.1?report=genbank&log$=protalign&blast_rank=16&RID=881ZAR9G015) | *Legionella gratiana NCTC12388* | 40 | Lb3ssB |
| Lb2ssB | Legionella taurinensis GCMI-01 | PUT41642.1 | [WP_058463547.1](https://www.ncbi.nlm.nih.gov/protein/WP_058463547.1?report=genbank&log$=protalign&blast_rank=27&RID=881ZAR9G015) | *Legionella cincinattinsis NCTC12438* | 35 | Lb3ssB |
| Lb2ssB | Legionella taurinensis GCMI-01 | PUT41642.1 | [WP_128129933.1](https://www.ncbi.nlm.nih.gov/protein/WP_128129933.1?report=genbank&log$=protalign&blast_rank=28&RID=881ZAR9G015) | *Legionella sainthelensi NCTC12450* | 36 | Lb3ssB |
| Lb2ssB | Legionella taurinensis GCMI-01 | PUT41642.1 | [WP_003631686.1](https://www.ncbi.nlm.nih.gov/protein/WP_003631686.1?report=genbank&log$=protalign&blast_rank=25&RID=881ZAR9G015) | *Legionella longbeachae* NSW150 | 36 | Lb3ssB |
| Lb2ssB | Legionella taurinensis GCMI-01 | PUT41642.1 | [WP_045106094.1](https://www.ncbi.nlm.nih.gov/protein/WP_045106094.1?report=genbank&log$=protalign&blast_rank=8&RID=881ZAR9G015) | *Legionella hackeliae NCTC11979* | 74 | Lb2ssB |
| Lb2ssB | Legionella taurinensis GCMI-01 | PUT41642.1 | [WP_058450193.1](https://www.ncbi.nlm.nih.gov/protein/WP_058450193.1?report=genbank&log$=protalign&blast_rank=9&RID=881ZAR9G015) | *Legionella jamestowniensis DSM 19215* | 77 | Lb2ssB |
| Lb2ssB | Legionella taurinensis GCMI-01 | PUT41642.1 | [WP_058471062.1](https://www.ncbi.nlm.nih.gov/protein/WP_058471062.1?report=genbank&log$=protalign&blast_rank=12&RID=881ZAR9G015) | *Legionella jordanis NCTC11533* | 66 | Lb2ssB |
| Lb2ssB | Legionella taurinensis GCMI-01 | PUT41642.1 | [KTC95114.1](https://www.ncbi.nlm.nih.gov/protein/KTC95114.1?report=genbank&log$=protalign&blast_rank=6&RID=881ZAR9G015) | *Legionella feeleii NCTC11978* | 75 | Lb2ssB |
| Lb2ssB | Legionella taurinensis GCMI-01 | PUT41642.1 | [WP_043874069.1](https://www.ncbi.nlm.nih.gov/protein/WP_043874069.1?report=genbank&log$=protalign&blast_rank=1&RID=88617NWR015) | *Legionella massiliensis LegA* | 39 | Lb3ssB |
| Lb2ssB | Legionella taurinensis GCMI-01 | PUT41642.1 | [WP_058496374.1](https://www.ncbi.nlm.nih.gov/protein/WP_058496374.1?report=genbank&log$=protalign&blast_rank=1&RID=8864U05V014) | *Legionella drozankii* LLAP-1 | 35 | Lb3ssB |
| Lb2ssB | Legionella taurinensis GCMI-01 | PUT41642.1 | [WP_058503271.1](https://www.ncbi.nlm.nih.gov/protein/WP_058503271.1?report=genbank&log$=protalign&blast_rank=31&RID=881ZAR9G015) | *Legionella nautarum ATCC 49506* | 35 | Lb3ssB |
| Lb2ssB | Legionella taurinensis GCMI-01 | PUT41642.1 | [WP_126337699.1](https://www.ncbi.nlm.nih.gov/protein/WP_126337699.1?report=genbank&log$=protalign&blast_rank=19&RID=881ZAR9G015) | *Legionella spiritensis NCTC12082* | 39 | Lb3ssB |
| Lb2ssB | Legionella taurinensis GCMI-01 | PUT41642.1 | [WP_058525697.1](https://www.ncbi.nlm.nih.gov/protein/WP_058525697.1?report=genbank&log$=protalign&blast_rank=1&RID=886DBDCJ014) | *Legionella erythra SE-32A-C8* | 93 | Lb2ssB |
| Lb2ssB | Legionella taurinensis GCMI-01 | PUT41642.1 | [WP_082651485.1](https://www.ncbi.nlm.nih.gov/protein/WP_082651485.1?report=genbank&log$=protalign&blast_rank=2&RID=881ZAR9G015) | *Legionella rubrilucens WA-270A-C2* | 98.68 | Lb2ssB |
| Lb2ssB | Legionella taurinensis GCMI-01 | PUT41642.1 | [WP_028385894.1](https://www.ncbi.nlm.nih.gov/protein/WP_028385894.1?report=genbank&log$=protalign&blast_rank=26&RID=881ZAR9G015) | *Legionella geestiana* NCTC12373 | 35 | Lb3ssB |
| Lb2ssB | Legionella taurinensis GCMI-01 | PUT41642.1 | [WP_058524258.1](https://www.ncbi.nlm.nih.gov/protein/WP_058524258.1?report=genbank&log$=protalign&blast_rank=17&RID=881ZAR9G015) | *Legionella birminghamensis NCTC12437* | 39 | Lb3ssB |
| Lb2ssB | Legionella taurinensis GCMI-01 | PUT41642.1 | [WP_058506864.1](https://www.ncbi.nlm.nih.gov/protein/WP_058506864.1?report=genbank&log$=protalign&blast_rank=18&RID=881ZAR9G015) | *Legionella quinlivani NCTC12433* | 40 | Lb3ssB |
| Lb2ssD | Legionella taurinensis GCMI-01 | PUT41641.1 | [WP_058473477.1](https://www.ncbi.nlm.nih.gov/protein/WP_058473477.1?report=genbank&log$=protalign&blast_rank=10&RID=88N25TD5014) | *Legionella quateirensis NCTC12376* | 35 | Lb3ssD |
| Lb2ssD | Legionella taurinensis GCMI-01 | PUT41641.1 | [WP_045094813.1](https://www.ncbi.nlm.nih.gov/protein/WP_045094813.1?report=genbank&log$=protalign&blast_rank=8&RID=88N25TD5014) | *Legionella fallonii LLAP-10* | 67 | Lb2ssD |
| Lb2ssD | Legionella taurinensis GCMI-01 | PUT41641.1 | [WP_058481064.1](https://www.ncbi.nlm.nih.gov/protein/WP_058481064.1?report=genbank&log$=protalign&blast_rank=20&RID=88N25TD5014) | *Legionella waltersii NCTC13017* | 30.38 | NHLP-type T1SS MFP |
| Lb2ssD | Legionella taurinensis GCMI-01 | PUT41641.1 | [WP_083502897.1](https://www.ncbi.nlm.nih.gov/protein/WP_083502897.1?report=genbank&log$=protalign&blast_rank=22&RID=88N25TD5014) | *Legionella gratiana NCTC12388* | 26 | Lb3ssD |
| Lb2ssD | Legionella taurinensis GCMI-01 | PUT41641.1 | [WP_083502697.1](https://www.ncbi.nlm.nih.gov/protein/WP_083502697.1?report=genbank&log$=protalign&blast_rank=28&RID=88N25TD5014) | *Legionella cincinattinsis NCTC12438* | 25 | Lb3ssD |
| Lb2ssD | Legionella taurinensis GCMI-01 | PUT41641.1 | [VEH33247.1](https://www.ncbi.nlm.nih.gov/protein/VEH33247.1?report=genbank&log$=protalign&blast_rank=32&RID=88N25TD5014) | *Legionella sainthelensi NCTC11988* | 27 | Lb3ssD |
| Lb2ssD | Legionella taurinensis GCMI-01 | PUT41641.1 | [CBJ11904.1](https://www.ncbi.nlm.nih.gov/protein/CBJ11904.1?report=genbank&log$=protalign&blast_rank=30&RID=88N25TD5014) | *Legionella longbeachae* NSW150 | 25 | Lb3ssD |
| Lb2ssD | Legionella taurinensis GCMI-01 | PUT41641.1 | [WP_045106093.1](https://www.ncbi.nlm.nih.gov/protein/WP_045106093.1?report=genbank&log$=protalign&blast_rank=6&RID=88N25TD5014) | *Legionella hackeliae NCTC11979* | 77 | Lb2ssD |
| Lb2ssD | Legionella taurinensis GCMI-01 | PUT41641.1 | [WP_058450194.1](https://www.ncbi.nlm.nih.gov/protein/WP_058450194.1?report=genbank&log$=protalign&blast_rank=7&RID=88N25TD5014) | *Legionella jamestowniensis DSM 19215* | 74 | Lb2ssD |
| Lb2ssD | Legionella taurinensis GCMI-01 | PUT41641.1 | [WP_058471063.1](https://www.ncbi.nlm.nih.gov/protein/WP_058471063.1?report=genbank&log$=protalign&blast_rank=9&RID=88N25TD5014) | *Legionella jordanis NCTC11533* | 66 | Lb2ssD |
| Lb2ssD | Legionella taurinensis GCMI-01 | PUT41641.1 | [WP_115175933.1](https://www.ncbi.nlm.nih.gov/protein/WP_115175933.1?report=genbank&log$=protalign&blast_rank=3&RID=88N25TD5014) | *Legionella feeleii NCTC11978* | 78 | Lb2ssD |
| Lb2ssD | Legionella taurinensis GCMI-01 | PUT41641.1 | [WP_043874070.1](https://www.ncbi.nlm.nih.gov/protein/WP_043874070.1?report=genbank&log$=protalign&blast_rank=2&RID=8G20F8PJ01R) | *Legionella massiliensis LegA* | 31 | Lb3ssD |
| Lb2ssD | Legionella taurinensis GCMI-01 | PUT41641.1 | [WP_083497953.1](https://www.ncbi.nlm.nih.gov/protein/WP_083497953.1?report=genbank&log$=protalign&blast_rank=1&RID=8G20F8PJ01R) | *Legionella drozankii* LLAP-1 | 28 | Lb3ssD |
| Lb2ssD | Legionella taurinensis GCMI-01 | PUT41641.1 | [WP_083503737.1](https://www.ncbi.nlm.nih.gov/protein/WP_083503737.1?report=genbank&log$=protalign&blast_rank=31&RID=88N25TD5014) | *Legionella nautarum ATCC 49506* | 27 | Lb3ssD |
| Lb2ssD | Legionella taurinensis GCMI-01 | PUT41641.1 | [WP_126337701.1](https://www.ncbi.nlm.nih.gov/protein/WP_126337701.1?report=genbank&log$=protalign&blast_rank=17&RID=88N25TD5014) | *Legionella spiritensis NCTC12082* | 33 | Lb3ssD |
| Lb2ssD | Legionella taurinensis GCMI-01 | PUT41641.1 | [WP_058525696.1](https://www.ncbi.nlm.nih.gov/protein/WP_058525696.1?report=genbank&log$=protalign&blast_rank=1&RID=8G25B88V015) | *Legionella erythra SE-32A-C8* | 95 | Lb2ssD |
| Lb2ssD | Legionella taurinensis GCMI-01 | PUT41641.1 | [WP_058531948.1](https://www.ncbi.nlm.nih.gov/protein/WP_058531948.1?report=genbank&log$=protalign&blast_rank=2&RID=88N25TD5014) | *Legionella rubrilucens WA-270A-C2* | 97 | Lb2ssD |
| Lb2ssD | Legionella taurinensis GCMI-01 | PUT41641.1 | [WP_028385893.1](https://www.ncbi.nlm.nih.gov/protein/WP_028385893.1?report=genbank&log$=protalign&blast_rank=23&RID=88N25TD5014) | *Legionella geestiana* NCTC12373 | 27 | Lb3ssD |
| Lb2ssD | Legionella taurinensis GCMI-01 | PUT41641.1 | [WP_083503162.1](https://www.ncbi.nlm.nih.gov/protein/WP_083503162.1?report=genbank&log$=protalign&blast_rank=26&RID=88N25TD5014) | *Legionella birminghamensis NCTC12437* | 33 | Lb3ssD |
| Lb2ssD | Legionella taurinensis GCMI-01 | PUT41641.1 | [SEG09786.1](https://www.ncbi.nlm.nih.gov/protein/SEG09786.1?report=genbank&log$=protalign&blast_rank=11&RID=88N25TD5014) | *Legionella quinlivani NCTC12433* | 32 | Lb3ssD |

References

1. Smith TJ, Sondermann H, O’Toole GA. Type 1 Does the Two-Step: Type 1 Secretion Substrates with a Functional Periplasmic Intermediate. J Bacteriol. 2018;200:JB.00168-18. doi:10.1128/JB.00168-18.

2. Smith TJ, Font ME, Kelly CM, Sondermann H, O’toole GA 4. An N-terminal Retention Module Anchors the Giant Adhesin LapA of Pseudomonas 1 fluorescens at the Cell Surface: A Novel Sub-family of Type I Secretion Systems 2 3 Downloaded from. J Bacteriol. 2018. doi:10.1128/JB.00734-17.
